# Synthesis, Characterization and Biological Evaluation of Novel Benzamidine Derivatives: Newer Antibiotics for Periodontitis Treatment

**DOI:** 10.3390/antibiotics11020207

**Published:** 2022-02-07

**Authors:** Mohammad Auwal Sa’ad, Ramasamy Kavitha, Shivkanya Fuloria, Neeraj Kumar Fuloria, Manickam Ravichandran, Pattabhiraman Lalitha

**Affiliations:** 1 Department of Biochemistry, Faculty of Medicine, AIMST University, Bedong 08100, Kedah, Malaysia; wurosam@gmail.com; 2 Centre of Excellence for Vaccine Development (CoEVD), Faculty of Applied Science, AIMST University, Bedong 08100, Kedah, Malaysia; 3 Department of Biotechnology, Faculty of Applied Science, AIMST University, Bedong 08100, Kedah, Malaysia; rkavitha_78@yahoo.com; 4 Centre of Excellence for Biomaterials Engineering, Faculty of Pharmacy, AIMST University, Bedong 08100, Kedah, Malaysia; shivkanya_fuloria@aimst.edu.my; 5 Center for Transdisciplinary Research, Department of Pharmacology, Saveetha Institute of Medical and Technical Sciences, Saveetha Dental College and Hospital, Saveetha University, Chennai 600077, India

**Keywords:** periodontitis, benzamidine, anti-bacterial agents, cytotoxicity, hydrazide, aldehydes, esters, imines

## Abstract

Periodontal disease (PD) is complex polymicrobial disease which destroys tooth-supporting tissue. Although various synthetic inhibitors of periodontitis-triggering pathogens have been recognized, their undesirable side effects limit their application. Hence, the present study intended to perform the synthesis, characterization, antimicrobial evaluation, and cytotoxicity analysis of novel benzamidine analogues (NBA). This study involved the synthesis of novel imino bases of benzamidine (**4a**–**c**), by reacting different aromatic aldehydes with 2-(4-carbamimidoylphenoxy) acetohydrazide (**3**), which was synthesized by the hydrazination of ethyl 2-(4-carbamimidoylphenoxy) acetate (**2**), the derivative of 4-hydroxybenzene carboximidamide (**1**). This was followed by characterization using FTIR, ^1^H, ^13^C NMR and mass spectrometry. All synthesized compounds were further tested for antimicrobial potential against PD-triggering pathogens by the micro broth dilution method. The cytotoxicity analysis of the NBA against HEK 293 cells was conducted using an MTT assay. The present study resulted in a successful synthesis of NBA and elucidated their structures. The synthesized NBA exhibited significant antimicrobial activity values between 31.25 and 125 µg/mL against tested pathogens. All NBA exhibited weak cytotoxicity against HEK 293 cells at 7.81 µg, equally to chlorhexidine at 0.2%. The significant antimicrobial activity of NBA against PD-triggering pathogens supports their potential application in periodontitis treatment.

## 1. Introduction

Periodontitis is a chronic oral inflammatory disorder which causes the disruption of periodontal tissue and tooth loss [1]. Reports have suggested that the global burden of severe periodontal disease (PD) has increased substantially at a severe rate of 8.44% from 1990 to 2019 worldwide [2]. The oral microbiome contains diverse species of bacteria, with red complex microorganisms responsible for PD [3]. The oral cavity is known to harbor over 600 bacterial species [4], and the complex interaction between them causes periodontal disease. The use of antibiotics, for example, chlorhexidine and tetracyclines, is the treatment of choice for periodontitis [5]. However, antibiotics offers several disadvantages due to increasing evidence showing that bacteria are becoming more resistant to antibiotics [6]. Recently, imino analogues and benzamidines have become a subject of interest for many researchers due to their antimicrobial and antifungal activities [7,8,9,10]. Imines are reported to exhibit a broad range of biological activities, including antibacterial activity [11]. The antimicrobial assay results of a previous study indicate that the majority of the synthesized derivatives exhibited promising antimicrobial activity [12]. With regard to antibacterial compounds, the imines and their analogues are reported to exhibit inhibition activity against Gram-positive and Gram-negative bacteria [13]. Ester compounds exhibit antimicrobial and antifungal inhibition activities against some Gram-positive and Gram-negative bacteria, having antifungal and antioxidant activity [14,15,16,17]. Evidence suggests hydrazide to have noteworthy antibacterial potential [18]. Some hydrazide–hydrazone analogues were demonstrated to have excellent inhibition properties against pathogenic organisms, with some having better antimicrobial activity when compared with known antibiotics [19,20]. Although several inhibitors have been developed, the global prevalence of PD is challenging. This creates a need for efficient and safe inhibitors of periodontitis. The literary evidence concerning the high antimicrobial potential and safety aspects of imines and benzamidines motivated the investigators to design the present study to develop some novel benzamidine analogues as effective and safe inhibitors of periodontitis.

## 2. Results and Discussion

### 2.1. Chemistry

**Scheme 1:**
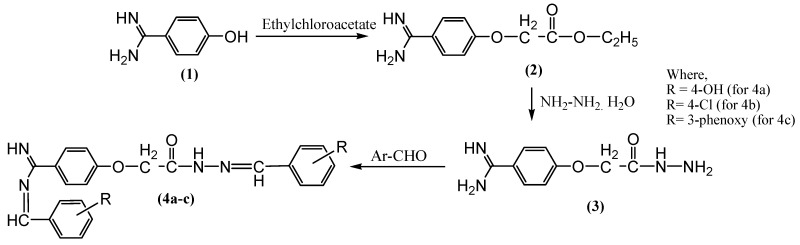
Synthesis of NBA.

The treatment of 4-hydroxybenzenecarboximidamide (**1**) with ethyl chloroacetate in anhydrous conditions produced ethyl-2-(4-carbamimidoylphenoxy)acetate (**2**) (Scheme 1). In this esterification reaction, the hydrogen from the hydroxyl group underwent replacement with the ethylacetate group of ethyl chloroacetate to form compound (**2**). Previous research reported that reacting hydroxybenzaldehyde with ethyl chloroacetate leads to the successful synthesis of cyclic esters [21]. Another study showed that reacting 3-aryl-1-(2-naphthyl)-prop-2-en-1-ones with ethyl chloroacetate using dry acetone results in successful ester synthesis, as in the present study. The melting point of compound (**2**) was found to be 42 °C, similar to that of esters synthesized from saturated fatty acids (methyl behenate) with a melting point of 41 °C [22].

The treatment of compound (**2**) with a hydrazine hydrate in the presence of absolute ethanol yielded 2-(4-carbamimidoylphenoxy) acetohydrazide (**3**) (Scheme 1). In this hydrazination/amination reaction, the compound (**2**) ethoxy group underwent replacement with a hydrazine hydrate group of hydrazides to yield compound (**3**) [23]. The treatment of resultant compound (**3**) with different aldehydes such as 4-hydroxybenzaldehyde, 4-chlorobenzaldehyde, and 3-phenoxy benzaldehyde yielded substituted imines (**4a**–**c**), respectively. In this Schiff’s reaction, the reflux of compound (**3**) with various aromatic aldehydes resulted in imines (**4a**–**c**) synthesis [24]. Schiff base synthesis by previous researchers resulted in the synthesis of imine bases, having physical characteristics similar to the compounds synthesized in the present study [25,26].

The Scheme 1 synthetic route offered intermediaries and final compounds in good yield. The synthesized compounds’ purity was determined based on their sharp melting point and single spot TLC pattern. The TLC (thin-layer chromatography) silica gel plate was carried out to verify the purity of the synthesized compound. The spotted compounds were visualized under the Sprectroline UV Viewing chamber with a wavelength of 254 nm and the results were recorded. The analysis of synthesized benzamidine analogues using FTIR, ^1^H, ^13^C-NMR, and mass spectrometric data confirmed the proposed structure of compounds. The successful synthesis of compound (**2**) was confirmed by the presence of the characteristic IR bands at 1670 (C=O) and 3130 (C-H), ^1^H-NMR signal at 1.16 (t, *J* = 4.5 Hz, 3H, CH_3_) and 3.20 (q, *J* = 4.5 Hz, 2H, CH_2_), ^13^C-NMR signal at 29 (CH_3_) and 50 (CH_2_), and a mass spectrum ion peak at 222. The successful synthesis of compound (**3**) was confirmed by the IR band at 1099 (N-N), the disappearance of the ^1^H-NMR signal at 1.16 and 3.20, the appearance of an extra ^1^H-NMR signal at 8.27 (brs, NH) and 8.33 (brs, NH_2_), the disappearance of the ^13^C-NMR signal at 29 (CH_3_) and 50 (CH_2_), and a mass spectrum with a parent ion peak at 208. The disappearance of the characteristic IR band at 1099, the appearance of an IR band at 3344 (OH), the ^1^H-NMR signal at 9.65 (s, CH=N), the ^13^C-NMR signal at 145 (N-N=C), and a mass spectrum exhibiting a parent ion peak at 416 confirmed the successful synthesis of compound (**4a**–**c**).

### 2.2. Biological Activity

#### 2.2.1. In Vitro Antibacterial Activity of Synthesized Compounds

In vitro antimicrobial analysis includes a variety of methods that determine the susceptibility of drugs, plant extracts, and other synthetic substances against microorganisms [27,28,29]. The present study used the micro broth dilution method to determine the inhibition susceptibility of synthesized compounds against periodontal pathogens such as *P. gingivalis* (ATCC 33277), *E. coli* (pHuLUC3), *S. aureus* (ATCC 29213), *S. epidermidis* (ATCC 12228), *P. aeruginosa* (ATCC 10145), and *S. pyogenes* (clinical strain). The present study observed that all synthesized compounds exhibited a minimum inhibition concentration (MIC) against tested pathogens, with the lowest concentration at 31.25µg/mL and highest concentration at 125 µg/mL (Table 1). Furthermore, some NBA have exhibited excellent minimal bactericidal concentration (MBC) against the tested pathogens, with some having a higher activity than the standard used in the study (Ampicillin against *S. epidermidis*, as shown in Table 2). Meanwhile, none of the NBA exhibited MBC against *S. pyogenes*. Moreover, compound (**2**) only exhibited MBC against *P. gingivalis* at 125 µg/mL, but not against other tested microbes.

The present study showed that compound (**2**) possesses growth inhibition activity against *P. gingivalis* at 62.5 µg/mL concentration (Table 1). The synthesized ester in this study exhibited better MIC against *S. aureus* (125 µg), *S. epidermidis* (52.08 µg), and *P. aeruginosa* (62.5 µg) than those synthesized from sugar esters by other researchers when tested against the same species [30]. Although esters have antibacterial properties, little of their activity is known when it comes to oral microbes. A previous study showed that fatty acid esters exhibit inhibition activity when tested against *P. gingivalis* [31]. The ester’s ability to inhibit *P. gingivalis* was further confirmed with the current study. In addition, other studies confirmed the antimicrobial property of esters against Gram-positive and Gram-negative bacteria [32,33,34]. This study revealed that compound (**2**) exhibits MBC against *P. gingivalis* but not against other tested microorganisms, suggesting that compound (**2**) has bacteriostatic activity against other tested pathogens in the present study (Table 2).

In the present study, compound (**3**) exhibited excellent inhibition activity against *P. gingivalis* with an MIC of 62.5 µg/mL (Table 1). Although the newly synthesized hydrazide showed a moderate MIC against all the tested pathogens, with the lowest concentration at 31.25 µg/mL and highest being 104 µg/mL (Table 1), it only displayed MBC against *P. gingivalis* and *S. epidermidis* (Table 2). Hydrazide moieties has demonstrated extensive antimicrobial inhibition, better than other known antibiotics [35]. They are also known to have antimicrobial activity against Gram-positive and Gram-negative bacteria, including methicillin-resistant *S. aureus* [36,37,38].

In recent years, imine bases demonstrated excellent antibacterial activities with some studies reporting that it possesses better inhibition activity than some commonly used antibiotics [39]. Although imine bases are continuously being evaluated for their antimicrobial properties against bacteria, there are fewer studies when it comes to *P. gingivalis* growth inhibition. The present study observed that novel imino bases of benzamidine (**4a**–**c**) exhibited excellent growth inhibition against *P. gingivalis* in comparison to other synthesized compounds. Previous studies showed that imine bases inhibit bacteria growth [40,41,42]. A similar observation was noted in this study. In the present study, imino bases of benzamidine (**4a** and **4b**) are found to exhibit a better inhibition activity against *P. aeruginosa* than the standard antibiotic used as a control (Table 2). Although numerous studies have been conducted on the effect of plant extract against *P. gingivalis,* these newly synthesized compounds are more potent as compared with some of the plant’s extracts, synthetic substances, and other inhibitors [43,44,45,46]. All synthesized compounds in the present study exhibited an MIC against tested pathogens and revealed the potent activity of NBA to inhibit the growth of microorganisms.

#### 2.2.2. Cytotoxicity Analysis of Synthesized Compounds

**Figure 1:**
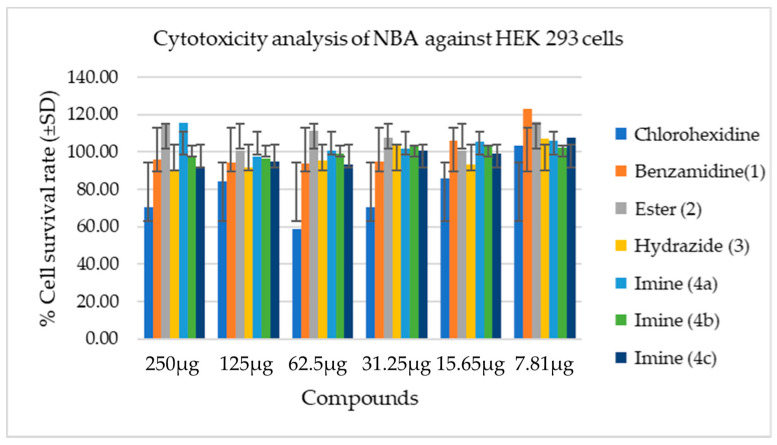
Cytotoxicity analysis of NBA against HEK 293 cells.

To determine cell viability, the MTT assay was used in the present study. Although all the NBA displayed low cytotoxicity against HEK 293 cells, ester (**2**) and imine (**4a**) displayed the least toxicity in all the concentrations with *p* < 0.05. Among all synthesized compounds, compound (**3**) yielded higher cytotoxicity. However, none of these synthesized compounds can be considered toxic when compared with chlorhexidine (control) due to their known low inhibition activity against healthy cells (Figure 1 and Table 3). Although esters are known to exhibit biological activities, there are fewer studies on their toxicity against healthy cells. Nevertheless, a study reported that allyl esters exhibit good tolerance against healthy CD-1 mice cells [47]. Similarly, in the present study, ester (**2**) showed the least cytotoxicity when tested against HEK 293 cells (Figure 1). On the contrary, another study reported that when ester derivatives are exposed to HK-2 cells at a high concentration, they exhibit mild but significant toxicity, and affect the cell metabolic activity [48]. In the present study, no significant toxicity was observed for the newly synthesized compound over HEK cells. This supports the safety aspect of the new compound. Hydrazide has tremendous biological activities [49,50,51], yet little is known about hydrazide toxicity against healthy cells. In this study, it was observed that hydrazide exhibits higher toxicity at a high concentration compared with other NBA. However, synthesized hydrazide showed no cytotoxicity at its lowest concentration (7.81 µg). The results of toxicity analysis in this study are also supported by the previous study [52]. The present research claims that compound (**4a**) exhibits the least toxicity at a higher concentration (250 µg), whereas compound (**4c**) exhibits high toxicity at a higher concentration (250 µg) in comparison with compounds (**4a** and **4b**) (Figure 1). However, overall, the imine bases exhibit low toxicity. The findings of the previous study also support the results of the present study [53].

Cell viability after exposure to different concentrations of samples using MTT assay. This figure shows the cell survival rate after exposing them to different concentrations of compounds (250–7.81 µg) and control (4–0.2%) after 24 hrs of incubation. The values obtained are expressed as mean ± SD (*n* = 3). 

Chlorhexidine is widely used as a mouth wash because of its anti-plaque activity. It has been suggested that 0.2% is relatively safe to use, although it may cause teeth staining [54,55]. In this study, chlorhexidine was used as a control at its lowest concentration (0.2%), and it exhibited an absence of cytotoxicity in contrast to compounds (**3**), (**4b**), and (**4c**), with a mild difference in their highest concentration (250 µg). However, compounds (**2**) and (**4a**) exhibited higher cell survival at their highest concentration (250 µg) when compared with chlorhexidine at its lowest concentration (0.2%). This indicates that novel benzamidine analogues exhibit no cytotoxicity when tested against HEK cells.

The successful results obtained from this study will pave the way for the manufacture of larger quantities of benzamidine analogues. The significance of this study is the ability of NBA to inhibit the growth of both Gram-positive and Gram-negative bacteria, which will hinder the progression of periodontitis and hence treat oral diseases and other associated conditions while displaying no toxicity at the in vitro stage. The resultant data from this study can serve as pre-clinical data for clinical trials and the commercialization of the synthesized compounds.

## 3. Materials and Methods

### 3.1. General Information

Chemicals, solvents and reagents used for synthesis were acquired from Merck KGaA (Darmstadt, Germany), Sigma-Aldrich Co. (St. Louis, MO, USA), Friendemann Schmidt Chemical, Washington, DC, USA, HmbG^®^ Chemicals, Hamburg, Germany, and Qrec Chemicals, Rawang, Malaysia. For filtration, Ashless Whattman No. 1 filter paper was used. Open capillary tube method was used to verify the newly synthesized compound’s melting point using SMP11 Analogue apparatus. With regard to the characterization of compounds, nuclear magnetic resonance spectra (NMR) were recorded on a ^1^H-NMR, and ^13^C-NMR (NMR 700 MHz ASCEND™ spectrometer) using deuterated DMSO solvent, on a δ value scale as the downfield chemical shift in ppm against tetramethylsilane (TMS). The NMR signals are stated as: s, single; d, doublet; t, triplet; m, multiplet; number of protons; and coupling constants (*J* value) in Hertz. Jasco ft/ir-6700 instrument was used to record the infrared spectra (IR) of compounds in a wavelength range of 400 to 4000 cm^−1^. The analysis of mass spectra was recorded from Direct Infusion IonTrap MS Full Scan (Thermo Scientific Q Exactive HF-X hybrid quadrupole-Orbitrap mass spectrometer, Waltham, MA, USA). Elemental analysis was performed on a Perkin Elmer 240 B and 240 C. Elemental analysis (C,H,N), indicated by employing element symbols, was within ±0.4% of theoretical values. The purity of compounds and monitoring of reactions were assessed by TLC on aluminum sheets with silica gel 60 F254 (0.2 mm) (Merck Millipore, Darmstadt, Germany) using methanol:acetone (9:1) as a solvent system in a UV chamber using a SPRECTROLINE^®^ CM-26 UV viewing chamber. The 4-hydroxybenzenecarboximidamide analogues were synthesized as per the protocol given by previous authors [25,56].

### 3.2. Synthesis

#### 3.2.1. Synthesis of Ethyl 2-(4-Carbamimidoylphenoxyacetate (**2**)

An equal molar concentration (0.01 mol) of 4-hydroxybenzenecarboximidamide, ethyl chloroacetate, and anhydrous potassium carbonate was dissolved in absolute ethanol. The mixture was refluxed for 18 h at 60 °C in a 500 mL round bottom flask using a heating mantle. The resulting mixture was filtered using Whatman filter paper. The compound obtained was recrystallized using absolute ethanol and activated charcoal to remove color impurities. It was filtered and dried to yield the pure compound (**2**). 

White crystalline (Yield 78%, m.p. 42 °C); IR (KBr, cm^−1^): 3265 (N-H), 3130 (=C-H), 2835 (C-H), 1670 (C=O), C=N (1608), 1487, 1527 (C=C); ^1^H-NMR (DMSO-d_6_): δ 1.16 (t, *J* = 4.5 Hz, 3H, CH_3_), 3.20 (q, *J* = 4.5 Hz, 2H, CH_2_), 4.88 (s, 2H, O-CH2-CO), 6.71–7.76 (m, 4H, Ar-H), 8.49 (s, 1H, C=NH), 8.98 (brs, NH_2_). ^13^C-NMR (DMSO-d_6_): δ 29 (CH_3_), 50 (O-CH2), 48 (CH2), 115, 117, 129, 131 (Ar-C), 164 (C=N), 173 (C=O). Mass (m/z): 222 (parent ion peak) and 223 (isotope peak); Anal. Calcd. for C_11_H_14_N_2_O_3_: C, 59.45; H, 6.35; N, 12.60; O, 21.60. Found: H, 6.42; C, 59.55; N, 12.53.

#### 3.2.2. Synthesis of 2-(4-Carbamimidoylphenoxy) Acetohydrazide (**3**)

An equimolar concentration of hydrazine hydrate and compound (**2**) (0.01 mol) was weighed and dissolved in absolute ethanol. Using a round bottom flask, the mixture was refluxed for at 55 °C for 8 h in anhydrous condition and filtered using filter paper. The compound was recrystallized to yield compound (**3**). 

Orange solid (Yield 82%, m.p. 52 °C); IR (KBr, cm^−1^): 3267 (N-H stretch), 3136 (Aromatic C-H stretch), 2966, 2852 (alkyl C-H stretch), C=O (1668), 1608 (C=N), 1489, 1438 (C=C), 1099 (N-N). ^1^H-NMR (DMSO-d_6_): δ 4.8 (s, 2H, O-CH2), 6.75–7.76 (m, 4H, Ar-H), 8.27 (brs, NH), 8.33 (brs, NH_2_), 8.41 (1H, C=NH), 8.90 (C-NH2). ^13^C-NMR (DMSO-d_6_): δ 48 (O-CH_2_), 115, 117, 129, 131, (Ar-C), 163 (C=N), 166 (C=O). Mass (m/z): 208 (parent ion peak) and 209 (isotope peak); Anal. Calcd. for C_9_H_12_N_4_O_2_: C, 51.92; H, 5.81; N, 26.91. C, 51.83; H, 5.87; N, 26.98.

#### 3.2.3. General Synthesis Procedure for the of N′-(Substituted Benzylidene)-2-(4-(N-(4-Hydroxybenzylidene)carbamimidoyl)phenoxy)acetohydrazide (**4a**–**c**)

The compound (**3**) (0.01 mol), aromatic aldehyde (0.01 mol) was weighed and dissolved into absolute ethanol. A 0.1 mL of glacial acetic acid was added into the mixture to serve as a catalyst. The mixture was refluxed at 50 °C for 8 h. After the completion of the reaction, the resultant mixture (imino bases) was dried to obtain the crude product. The crude was recrystallized using absolute ethanol and activated charcoal to offer pure compound **4a**. Similarly, the other compounds (**4b** and **4c**) were synthesized and subjected to TLC and CHN analysis to determine their purity. 

*N′-(4-hydroxybenzylidene)-2-(4-(N-(4-hydroxybenzylidene)carbamimidoyl)phenoxy)acetohydrazide* (**4a**). Brown crystalline (Yield 78%, m.p. 202 °C); IR (KBr, cm^−1^): 3344 (O-H), 2966 (C-H), C=N (1599), 1512, 1450 (C=C aromatic ring); ^1^H-NMR (DMSO-*d*_6_): δ 4.8 (s, 2H, O-CH2), 5.9 (s, 2H, OH), 6.66–7.67 (m, 12H, Ar-H), 7.99 (s, 1H, NH-N), 8.44 (s, 2H, C=NH), and 9.65 (s, CH=N); 13C-NMR: ^13^C-NMR (DMSO-d_6_): δ 62 (O-CH_2_), 115, 117, 129, 131, (Ar-C), 145 (N-N=C), 163 (C=NH), 164 (N=C), 166 (C=O); mass (m/z): 416 (parent ion peak) and 417 (isotope peak); Anal. Calcd. for C_23_H_20_N_4_O_4_: C, 66.34; H, 4.84; N, 13.45. Found: C, 66.49; H, 4.92; N, 13.51.

*N′-(4-chlorobenzylidene)-2-(4-(N-(4-chlorobenzylidene)carbamimidoyl)phenoxy)acetohydrazide* (**4b**). Brown crystalline (Yield 82%, m.p. 200 °C); IR (KBr, cm^−1^): 3095 (=C-H), 2987 (C-H), 1593 (C=N), 1512, 1489 (C=C aromatic ring); ^1^H-NMR (DMSO-*d*_6_): δ 4.8 (s, 2H, O-CH2), 6.80–7.88 (m, 12H, Ar-H), 7.98 (s, 1H, NH-N), 8.45 (s, 2H, C=NH), and 9.65 (s, CH=N); 13C-NMR: ^13^C-NMR (DMSO-d_6_): δ 48 (O-CH_2_), 115, 117, 129, 131, (Ar-C), 145 (N-N=C), 163 (C=NH), 164 (N=C), 166 (C=O); mass (m/z): 452 (parent ion peak) and 454 (isotope peak); Anal. Calcd. for C_23_H_18_Cl_2_N_4_O_2_: C, 60.94; H, 4.00; N, 12.36. Found: C, 60.82; N, 12.41; H, 4.05.

*N′-(3-phenoxybenzylidene)-2-(4-(N-(3-phenoxybenzylidene)carbamimidoyl)phenoxy)acetohydrazide* (**4c**). Brown crystalline (Yield 75%, m.p. 126 °C); IR (KBr, cm^−1^): 2962 (C-H), C=N (1583), 1485, 1448 (C=C aromatic ring); ^1^H-NMR (DMSO-*d*_6_): δ 4.8 (s, 2H, O-CH2), 6.80–7.88 (m, 12H, Ar-H), 7.99 (s, 1H, NH-N), 8.45 (s, 2H, C=NH), and 9.65 (s, CH=N); 13C-NMR: ^13^C-NMR (DMSO-d_6_): δ 48 (O-CH_2_), 115, 117, 129, 131, (Ar-C), 145 (N-N=C), 163 (1H, C=NH), 164 (N=C), 166 (C=O); mass (m/z): 568 (parent ion peak) and 569 (isotope peak); Anal. Calcd. for C_35_H_28_N_4_O_4_: C, 73.93; H, 4.96; N, 9.85. Found: C, 73.82; H, 4.89; N, 9.93.

### 3.3. Determination of Antimicrobial Activity

#### 3.3.1. General Information

To culture *P. gingivalis*, blood-enriched tryptic soy agar (eTSA) (Merck KGaA, Darmstadt, Germany) was prepared by dissolving 20 g of TSB, 15 g normal agar, and 5 g of yeast extract in 925 mL of deionized water. The mixture was stirred and adjusted to pH 7.4, and deionized water was then added to top it up to 1 L. The medium was autoclaved and cooled down to 50 °C. After it had cooled down, sterile filtered 5% L-cysteine (10 mL) (Bio-Basic, Markham, ON, Canada), dithiothreitol (10 mL) (Sigma Life Sciences, Burlington, MA, USA), 0.5 mg/mL vitamin K (Sigma Life Sciences, Burlington, MA, USA) and 50 mL of defibrinated sheep’s blood were aseptically added. After dispensing the agar into a sterile Petri dish, the plates were stored at 4 °C in airtight bags to avoid contamination. All agars were pre-incubated in the anaerobic atmosphere for overnight at 37 °C before plating out the bacteria. A broth microdilution in 96-well sterile plates was used to determine the minimum inhibition concentration (MIC) of *P. gingivalis* according to CLSI guidelines using eTSB broth. A two-fold serial dilution was carried out for all the NBA in triplicate, starting with the highest concentration at 125 µg/mL to the lowest concentration at 0.97 µg/mL. The antibiotic used here as control was ampicillin (Akum Drugs and Pharmaceuticals, New Delhi, India), with concentrations starting from 5 µg/mL to 0.03 µg/mL. All these dilutions were carried out aseptically. To inoculate bacterial culture, the density of the microorganism was adjusted to the density of 0.5 McFarland standard or 1−2 × 10^8^ CFU/mL [57]. To the microtiter plate, 100 µL of culture was aseptically added in all wells excluding the negative control, which contained only broth. The microtiter plates were then incubated in an anaerobic jar (Oxoid, Winchester, England) with a gas indicator (Thermo Fisher Scientific, Waltham, MA, USA) and a gas pack (Merck KGaA, Darmstadt, Germany) that generated 90% N_2_, 5% CO_2_ and H_2_ mixture for 46 h at 37 °C. 

Cation-adjusted Mueller–Hinton broth (CAMHB) and agar (CAMHA) (HiMedia, Mumbai, India) were used to culture *E. coli*, *S. aureus*, *S. epidermidis*, *S. pyogenes*, and *P. aeruginosa*, then incubated in aerobic conditions overnight before use. The MIC of *E. coli*, *S. aureus*, *S. epidermidis*, *S. pyogenes*, and *P. aeruginosa* was determined using the same method as that of *P. gingivalis*. However, the starting concentration for the known antibiotic control varies depending on the required concentration for each microorganism. In addition, Kanamycin (CSC Pharmaceuticals, Mumbai, India) was used for *P. aeruginosa* with a starting concentration of 375 µg/mL and the lowest concentration of 2.92 µg/mL. Furthermore, the microtiter plates were incubated at 37 °C for 18 h. After assessing the MIC of *P. gingivalis*, samples from the clear wells where there was no visible growth of the culture were aseptically plated on eTSB blood agar and eTSB agar, and incubated at 37 °C in an anaerobic jar with a gas indicator and gas pack with a composition of 90% N_2_, 5% CO_2_ and H_2_ mixture for 46 h. 

The minimum bactericidal concentration (MBC) of *S. pyogenes* was evaluated by plating the culture from the visible wells on CAMHA supplemented with 5% defibrinated blood. Other microorganisms, including *E. coli*, *S. aureus*, *S. epidermidis*, and *P. aeruginosa*, were plated on CAMHA and incubated at 37 °C for 18 h according to the guidelines given by CLSI [58]. After incubation, MBC was recorded as the lowest concentration of compound where there was no visible growth of the microorganism with agar clarity, the same as that of the negative control. All experiments were performed in triplicate.

#### 3.3.2. Cell Viability Assay

The cultivation of DMEM (Dulbecco’s modified Eagle medium) supplemented with 5% fetal bovine serum (FBS) (Sigma life science, Burlington, MA, USA) and 1% antibiotic (GIBCO, Waltham, MA, USA) was used to culture human embryonic kidney cells (HEK 293 ATCC) and incubated at 37 °C, with 5% CO_2_, and relative humidity of about 95% (Heal Force/HF90, Hong Kong, China). The cultivation and the subsequent sub-culturing of cells and preservation were performed following the guidelines provided by ATCC.

#### 3.3.3. Cell Counting

The present study adapted the hemocytometer (Hirschmann Laborgerate, Darmstadt, Germany) cell counting technique. Cells were washed with PBS (First base, Axil Scientific, Singapore) and treated with trypsin (Sigma life science, Burlington, MA, USA) to detach them from the flask surface. Cells were then incubated at 37 °C before being trypsinized by adding DMEM media. One hundred microliters of cells was measured and added into 0.9 mL of 0.2% trypan blue (Sigma life science, Burlington, MA, USA) in a sterile 1.5 mL micro-centrifuge tube. Fifteen microliters of stained cells were loaded into the upper chamber of the hemocytometer, covered with microscopic cover glass, and the 15 μL balance was transferred into the lower chamber. Under the inverted microscope (Olympus/CK40-F200, Shinjuku, Japan), the viable cells were not stained by trypan blue whereas the dead cells were stained. Equations (1) and (2) present the formula to determine the total viable cells and number of live cells in original suspension:

(1) $$\mathrm{Total}\;\mathrm{number}\;\mathrm{of}\;\mathrm{viable}\;\mathrm{cells}\;=\;\frac{\mathrm{Total}\;\mathrm{number}\;\mathrm{of}\;\mathrm{cells}\;\mathrm{from}\;4\;\mathrm{grids}}{4\;\left(\mathrm{four}\;\mathrm{corner}\;\mathrm{squares}\right)}$$

(2) $$\begin{array}{c} \mathrm{Number}\;\mathrm{of}\;\mathrm{live}\;\mathrm{cells}\;\mathrm{in}\;\mathrm{original}\;\mathrm{suspension}\;=\;\mathrm{Total}\;\mathrm{number}\;\mathrm{of}\;\mathrm{viable}\;\mathrm{cells}\;\times \\ 10\;(\mathrm{Dilution}\;\mathrm{factor})\;\times \;10^{4}/\mathrm{mL} \end{array}$$

#### 3.3.4. Cell Treatment

Various concentrations of NBA were used to treat cells. The highest concentration was 250 μg and lowest was 7.81 μg (μg/mL). Similarly, different concentrations of chlorhexidine were also used as a control. After 24 h of incubation, an inverted microscope was used to examine cell condition before proceeding with cell treatment. Using a two-fold serial dilution method, cells were aseptically treated with the compounds. The compound concentration used was as follows: 250 μg, to 7.81 μg. Different concentrations of filtered sterilized chlorhexidine were also used, with the highest at 4% and lowest at 0.2% [55]. All cells were treated with NBA (in triplicate) at different concentrations, except for the control. After treating cells with inhibitors, the cells were incubated again for 24 h at 37 °C, with 5% CO_2_, and with a relative humidity of about 95%.

#### 3.3.5. 3-[4,5-Dimethylthiazol-2-yl]2,5-diphenyl Tetrazolium Bromide (MTT) Assay

Following the 24 h incubation of the cells with inhibitors, 20μL of MTT reagent (0.5 mg/mL) (Sigma life science, Burlington, MA, USA) in PBS was added to each well, including the control in the dark, and the plates were covered with aluminum foil paper and incubated at 37 °C for 4 h. After 4 h of incubation, the cells were treated with 100 μL of MTT detergent (DMSO) (Sigma life science, Burlington, MA, USA). After treating cells with DMSO (dimethyl sulfoxide), the cells were then covered with foil paper and incubated again for 1 h. After 1 h, cells were taken out of the incubator and gently shaken using very low rpm on a microplate shaker. The sample OD was measured at 570 nm with the reference of 630 nm using Infinite 200 PRO (Tecan Microplate Reader, Mannedorf, Switzerland). A triplicate experiment was carried out. Using the following formula given in Equation (3), the percentage (%) of cell viability was determined:

(3) $$\mathrm{Cell}\;\mathrm{Viability}\;\left(\%\right)\;=\;\frac{\mathrm{Sample}\;\mathrm{Absorbance}\;\left(\mathrm{Treated}\;\mathrm{cells}\right)}{\mathrm{Control}\;\mathrm{Absorbance}}\;\times \;100$$

#### 3.3.6. Statistical Analysis

In the present study, GraphPad Prism software version 5 (GraphPad Software, Inc., San Diego, CA, USA) was used to analyse cytotoxicity statistical data. One-way analysis of variance (ANOVA) and Tukey’s post hoc test with multiple comparisons were used to determine the source of significant difference between the groups.

## 4. Conclusions

Based on physical and chemical characterization, melting point, IR spectrum, mass and NMR data, the present study confirmed the successful synthesis of novel benzamidine analogues. It demonstrated that newly synthesized compounds cause significant growth inhibition against *P. gingivalis*, *E. coli*, *S. aureus*, *S. epidermidis*, *S. pyogenes*, and *P. aeruginosa* between 125 and 31.25 µg/mL. Although none of the newly synthesized compounds exhibited MBC against *S. pyogenes*, perhaps a higher concentration is required. Nevertheless, in this study, compound (**4a**) and (**4c**) exhibited better growth inhibition against *P. aeruginosa* than the standard antibiotic used. The significant antimicrobial activity of NBA against PD-triggering pathogens supports their potential application in the treatment of periodontitis. Furthermore, in vitro cytotoxicity analyses of newly synthesized compounds against HEK 293 cells exhibited moderate to absent toxicity, with none having a significant difference when compared with the standard control used. However, in vivo study is essential to further confirm these findings.

## Figures and Tables

**Table 1:** MIC values for NBA.

Compound (µg/mL)	Organisms/Strain					
	*P. gingivalis*	*E. coli*	*S. aureus*	*S. epidermidis*	*P. aeruginosa*	*S. pyogenes*
Compound **2**	62.50	55.55	125	83.33	62.50	125
Compound **3**	62.50	31.25	62.50	62.50	41.66	104
Compound **4a**	62.50	31.25	62.50	31.25	31.25	104
Compound **4b**	62.50	31.25	62.50	62.50	31.25	83.33
Compound **4c**	31.25	31.25	83.33	62.50	52	62.50
Benzamidine (**1**)	62.50	31.25	125	52	62.50	104.16
Ampicillin	0.15	15.62	7.81	31.25	-	7.81
Kanamycin	-	-	-	-	46.87	-

**Table 2:** MBC values for NBA.

Compound (µg/mL)	Organisms/Strain					
	*P. gingivalis*	*E. coli*	*S. aureus*	*S. epidermidis*	*P. aeruginosa*	*S. pyogenes*
Compound **2**	125	-	-	-	-	-
Compound **3**	125	-	-	125	-	-
Compound **4a**	125	-	125	62.50	125	-
Compound **4b**	125	-	125	125	-	-
Compound **4c**	62.50	125	-	-	125	-
Benzamidine (**1**)	125	-		-	-	-
Ampicillin	0.62	62.50	62.50	125	-	62.50
Kanamycin	-	-	-	-	187.50	-

**Table 3:** Cytotoxicity values for NBA.

Concentration	Chlorhexidine	Compound 1	Compound 2	Compound 3	Compound 4a	Compound 4b	Compound 4c
250 µg	70.23 ± 13.62	96.03 ±17.65 *	114.80 ± 10.81 ***	114.80 ± 10.81	115.74 ± 20.62 ***	97.27 ± 17.44 *	91.29 ± 15.47
125 µg	84.15 ±17.16	94.41 ± 9.93	100.71 ± 6.74	100.71 ± 6.74	97.66 ± 6.08	96.69 ± 17.14	94.59 ± 6.03
62.50 µg	58.81 ± 6.68	93.56 ± 10.72 *	111.10 ± 16.38 ***	111.10 ± 16.38 ***	100.74 ± 13.44 ***	99.20 ± 19.54 ***	93.04 ± 15.18 ***
31.25 µg	70.45 ± 17.57	94.83 ± 17.46 *	107.65 ± 29.99 **	107.65 ± 29.99	101.89 ± 11.23	102.85 ± 12.81	100.59 ± 13.36
15.65 µg	86.00 ± 18.64	105.89 ± 19.53	100.67 ± 29.35	100.67 ± 29.35 **	105.31 ± 17.71 *	103.30 ± 17.78 **	99.28 ± 18.53 *
7.81 µg	103.49 ± 18.04	122.87 ± 17.59	116.06 ± 19.78	116.06 ± 19.78	106.1 ± 26.76	102.09 ± 22.91	107.60 ± 23.88

Note: Data were expressed as mean ± standard deviation of the mean. Statistical significance is indicated by * *p* < 0.05, ** *p* < 0.01; *** *p* < 0.001.

## Data Availability

The data presented in this study are available on request from the corresponding author.
